# Uncovering the Pleomorphic Hyalinizing Angiectatic Tumor: A Case Series and Literature Review

**DOI:** 10.22034/ijp.2026.2075131.3565

**Published:** 2026-08-01

**Authors:** Jaydeep Nilkanthrao Pol, Manasi Gosavi, Rakhi Vikas Jagdale, Mahendra Atmaram Patil, Anand Bhosale

**Affiliations:** 1 Department of Surgical Pathology, Mahatma Gandhi Cancer Hospital, Miraj, Maharashtra, India; 2 Department of Pathology, KAHER’s Jawaharlal Nehru Medical College, Belagavi, Karnataka, India; 3 Department of Pathology, Shri Siddhivinayak Ganapati Cancer Hospital, Miraj, Maharashtra, India; 4 Krishna Vishwa Vidyapeeth, Malkapur, Karad district, Satara, Maharashtra, India; 5 Department of Pathology, D. Y. Patil University School of Medicine and Pushpalata D. Y. Patil Hospital, Ambi, Pune, Maharashtra, India

**Keywords:** Pleomorphic hyalinizing angiectatic tumor, fibrohistiocytic tumor, angiectatic tumor, soft tissue neoplasm, intermediate malignancy, pleomorphism, recurrence

## Abstract

**Background & Objective:**

Pleomorphic hyalinizing angiectatic tumor (PHAT) is a rare fibrohistiocytic neoplasm, classified by the World Health Organization (WHO) as a “neoplasm of uncertain behavior of connective or soft tissue” within the soft tissue and bone tumor group. A literature review via Scopus and PubMed revealed approximately 133 reported cases worldwide, affecting individuals aged 10 to 86 years, with a slight female predominance and wide anatomical distribution.

**Case Presentation:**

We report a series of 4 PHAT cases, the first from India and the fourth largest globally. The cases include a 37-year-old male with a large upper abdominal wall mass, a 46-year-old male with a forearm swelling, a 36-year-old female with a lower leg mass, and a 40-year-old male with a lower abdominal mass. Histopathology showed characteristic features, including ectatic vessels, hyalinization, and pleomorphic spindle cells.

**Conclusion:**

Given its rarity and histologic overlap with other tumors, increased awareness and accurate histopathological diagnosis of PHAT are essential.

## Introduction

Pleomorphic hyalinizing angiectatic tumor (PHAT), a rare neoplastic lesion, has been classified as a “neoplasm of uncertain behavior of connective or soft tissue” under World Health Organization (WHO) “Soft Tissue and Bone Tumors.”(1) It occurs across a wide age range of 10 to 86 years, with female preponderance. The most common site is the subcutaneous tissues of the lower extremities, with diverse sites being described in the approximately 133 cases reported in the international literature to date. Minimum morphological criteria required for diagnosis are as follows(2):

• Ectatic hyalinizing blood vessels

• Pleomorphic stromal cells

• No necrosis/rare mitotic figures (<1/50 HPF)

• Variable inflammatory cell infiltrate

• Infiltrative border

Despite its distinct pathological characteristics, PHAT continues to be underrecognized in routine practice. We describe here 4 cases of PHAT, along with a detailed systematic review of published cases sourced after a comprehensive literature search of the Scopus and PubMed databases. We would like to highlight here that this is the first and largest case series from India and the fourth largest case series in the international literature.

## Case reports

**Fig. 1:**
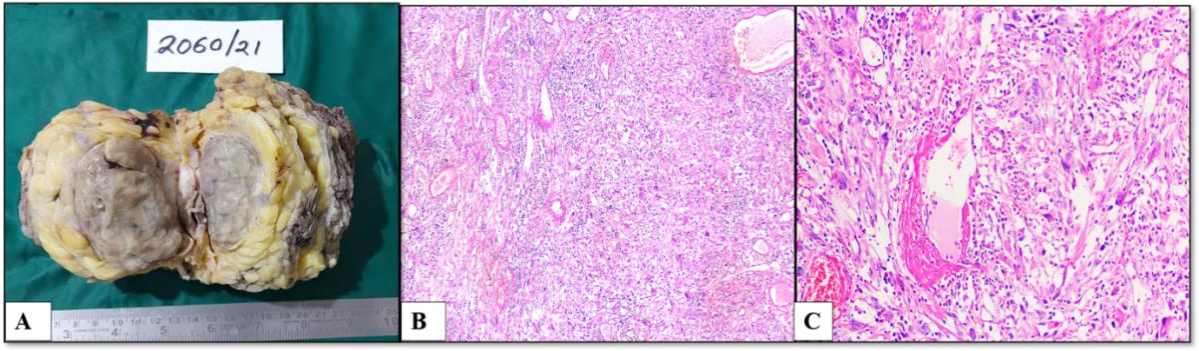
[Case 1]: (A) - Gray white well demarcated solid tumor with surrounding fibrofatty tissue. (B)- Spindle to pleomorphic tumor cells with scattered ectatic vessels, H&E, 10x. (C) Ectatic blood vessels with damaged hyalinized walls along with spindle cells & mixed inflammation; H&E, 20x

**Fig. 2:**
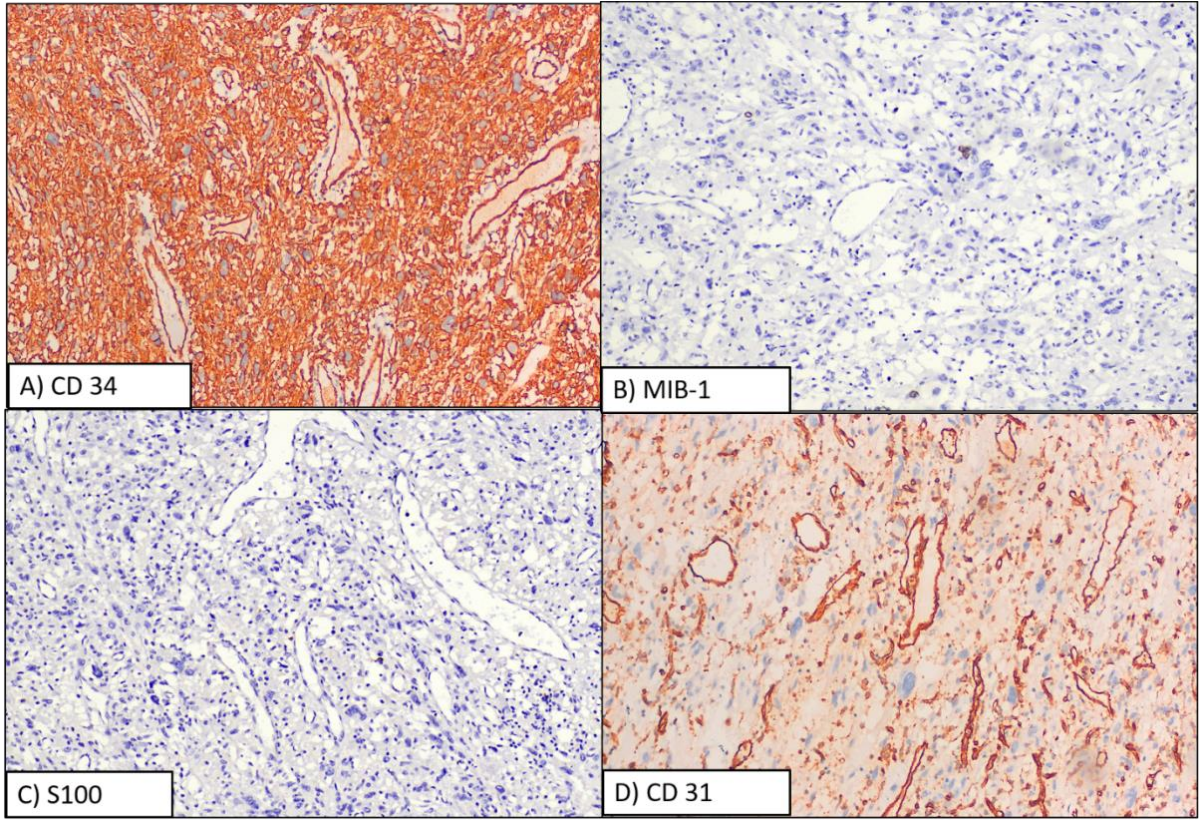
[Case 1]: Tumor cells express CD34, display low MIB-1 expression, are negative for S100 and variably and focally express CD31

Case 1: A 37-year-old male presented with a left upper anterior abdominal wall mass that had gradually increased in size over the last 2 years. Ultrasonography showed a well-circumscribed heterogeneous mass with areas of increased vascularity, based on which a clinical diagnosis of soft tissue tumor was made. The patient underwent local excision with wide margins. On gross examination, the tumor measured 7.5 × 7 × 5 cm. The cut section was fleshy and spongy (Fig 1A). On microscopy, a solid tumor composed of spindle and bizarre pleomorphic cells was seen, with numerous tumor giant cells (Fig 1B, 1C). Scattered ectatic blood vessels with damaged hyalinized walls were also evident (Fig 1C). A differential diagnosis of angiosarcoma and PHAT was given. Immunohistochemistry (IHC) was then performed, and the tumor cells were found to express membranous CD34 positivity. Nuclear Fli-1 and membranous podoplanin positivity were also seen. CD31 was variably and focally expressed. S100 was negative. Ki-67 was less than 5% (Fig 2). In view of the above features, a diagnosis of PHAT was preferred over angiosarcoma, considering the typical morphology, lack of mitosis despite the marked pleomorphism, and low Ki-67 labeling index. This patient has remained tumor-free for the last 3 years after excision of the tumor.

**Fig. 3:**
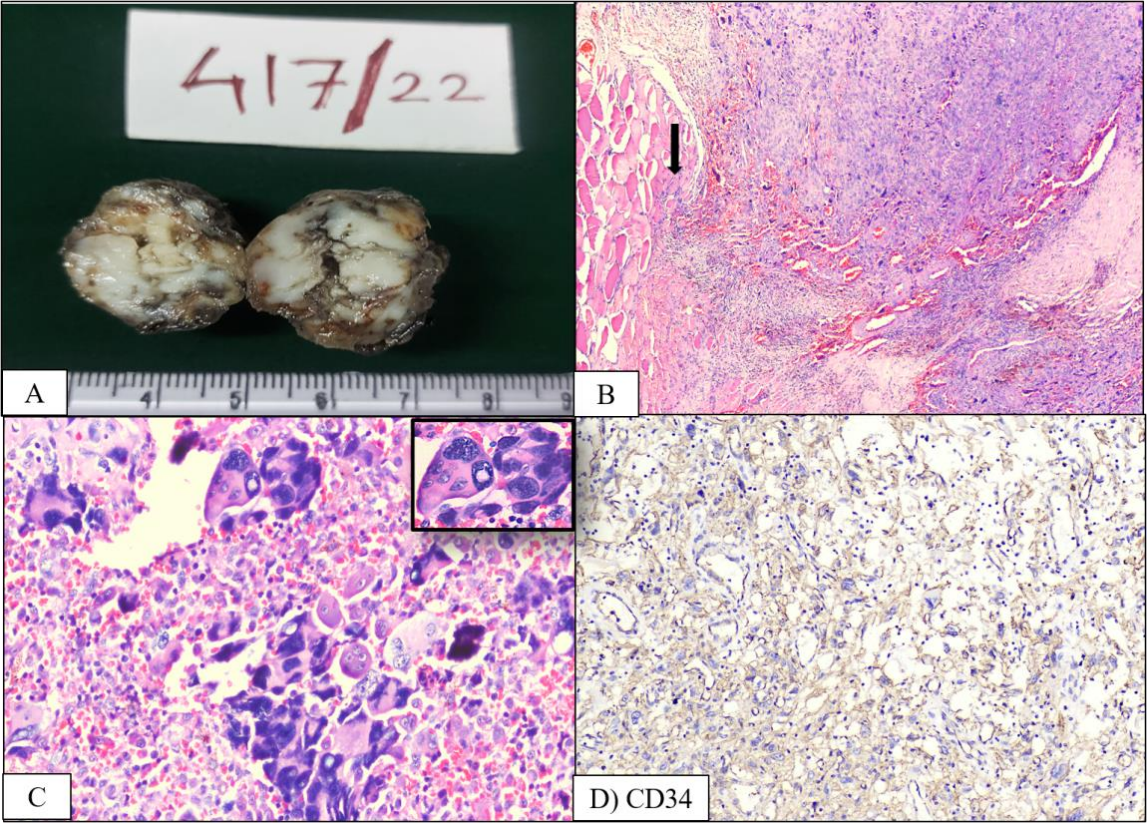
[Case 2]: (A)- tumor showing variegated appearance, few gray white areas admixed with dark brown hemorrhagic areas. (B)- Solid tumor with spindle and pleomorphic cells arranged in fascicles, locally infiltrative into surrounding muscle (arrow). H&E, 10x. (C)- Pleomorphic cells with large, atypical, hyperchromatic nuclei, prominent nucleoli and abundant eosinophilic cytoplasm, inset shows pseudoinclusions; H&E, 40x. (D)-Tumour cells showing CD34 expression.

Case 2: A 46-year-old male patient presented with swelling over the left forearm. With a clinical diagnosis of benign soft tissue tumor, excision was performed. On gross examination, the tumor was found to measure 2.8 × 2.5 × 2.4 cm. The cut section displayed a variegated appearance, with a few solid gray-white areas admixed with dark brown hemorrhagic areas (Fig 3A). On microscopy, a circumscribed tumor composed of spindle and pleomorphic cells arranged in fascicles showing local infiltration was seen (Fig 3B). Spindle cells had oval to elongated nuclei and displayed pleomorphism, with large, atypical, hyperchromatic nuclei, prominent nucleoli, and abundant eosinophilic cytoplasm. Intranuclear inclusions were noted (Fig 3C). A few large ectatic blood vessels with damaged hyalinized walls were seen. Many admixed osteoclastic giant cells were noted. Areas of hemorrhage and hemosiderin deposition were seen. The mitotic count was less than 1/10 HPF. No areas of necrosis were noted. A diagnosis of PHAT was given in view of the typical morphology. IHC showed membranous CD34 positivity, while S100 was negative (Fig 3D). In the 2-year follow-up, no recurrence of the tumor was seen in this patient.

**Fig. 4:**
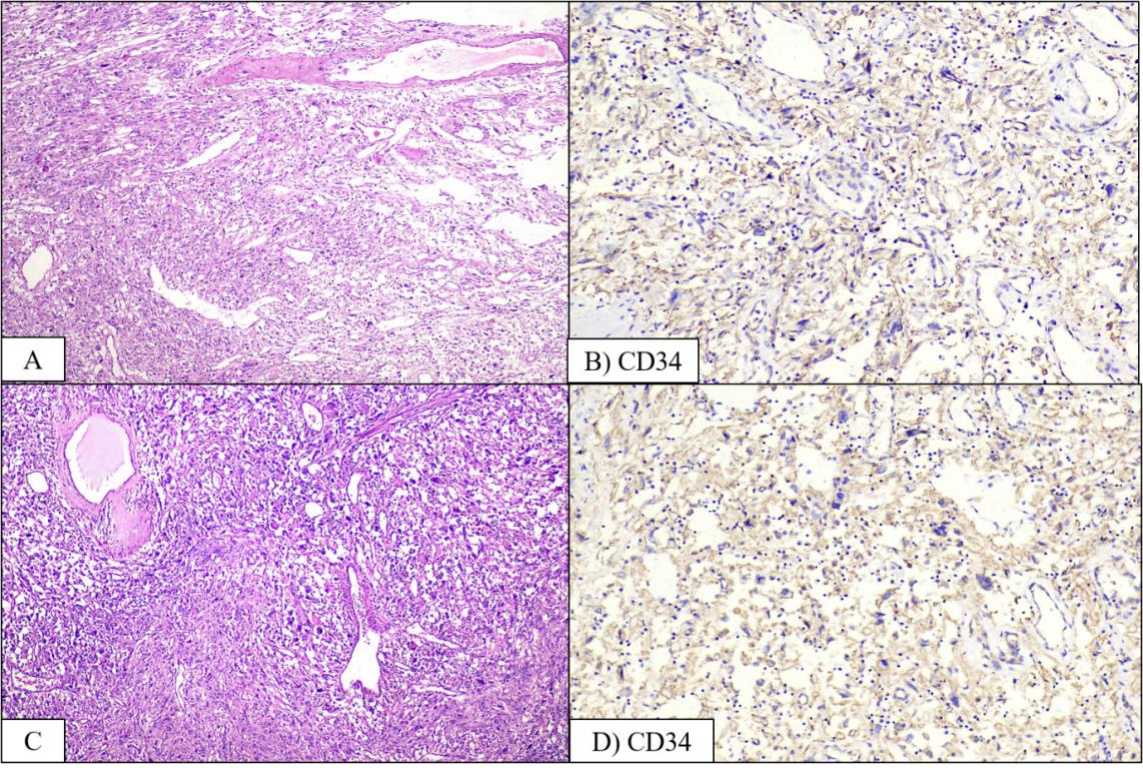
[Case 3] (A) Pleomorphic tumour cells with scattered hyalinized ectatic vessels; H&E, 20x; (B) showing CD34 expression. [Case 4] (C) Solid tumor with spindle and pleomorphic cells arranged in fascicles with ectatic blood vessels; H&E, 20x; (D)showing CD34 expression.

Case 3: A 36-year-old female patient presented with a slow-growing swelling over the lower leg for the last 3 years. On excision, the tumor was found to measure 4 × 3.5 × 3 cm. It was well circumscribed, with homogeneous grayish-yellow areas and areas of hemorrhage seen. Microscopy showed a well-circumscribed tumor composed of spindle cells arranged in short fascicles. Scattered pleomorphic cells with hyperchromatic nuclei and prominent nucleoli were seen. Ectatic blood vessels with hyalinized walls, areas of hemorrhage, and hemosiderin deposition were noted. Mitotic activity was infrequent (<1/10 HPF). IHC showed membranous CD34 positivity, while S100 was negative (Fig 4A and 4B). A diagnosis of PHAT was rendered. No recurrence of the tumor was noted on follow-up.

Case 4: A 40-year-old male patient presented with a left-sided lower abdominal mass. Ultrasonography revealed a large, well-circumscribed heterogeneous mass with mixed echogenicity and vascular proliferation, provisionally diagnosed as a soft tissue tumor. The patient opted for surgery with wide local excision. On examination, the tumor measured 9.6 × 7.4 × 4.4 cm and was well capsulated. The cut section showed a well-circumscribed mass with grayish-brown myxoid areas. Microscopy revealed an encapsulated tumor composed of hypo- and hypercellular areas of spindle to stellate to ovoid cells arranged in short whorls, bundles, and fascicles. Individual cells showed oval to spindle vesicular nuclei with occasional nucleoli and eosinophilic cytoplasm. Marked collagenization was seen. Numerous large ectatic, blood-filled spaces, along with small- to medium-sized blood vessels, were observed. Perivascular hyalinization was present. A chronic lymphocytic inflammatory infiltrate was noted, along with rare mitotic figures and mild nuclear atypia. The tumor was seen to have infiltrative margins. On IHC, tumor cells expressed CD34, Fli-1, and podoplanin and were negative for S100 (Fig 4C and 4D). The tumor was also negative for STAT6, ruling out solitary fibrous tumor. The MIB-1 labeling index was less than 1%. In view of the above features, a diagnosis of PHAT was given. The patient has not reported any recurrence in the last 3 and a half years since the diagnosis.

## Discussion

PHAT was first described in 1996 by Smith et al in a series of 14 cases, which is the second largest series described in the literature(3). The largest documented case series of 41 cases was reported by Folpe et al in 2004, while the third largest case series of 9 cases was reported by Qi Ke et al in 2007(1).

PHAT arises from a fibrocytic/fibrohistiocytic background. The tumor cells are of primitive uncommitted mesenchymal origin(4). Smith theorized that tumor cells intrude on the vessel walls, leading to endothelial damage and subsequent hyalinization of vessels. Mast cells further impact the vascular damage by increasing vascular permeability(5). However, Folpe and Weiss observed that vascular damage may represent an early change, occurring even in the absence of significant spindle cell proliferation. They further proposed that a myxoid spindle cell component at the periphery could be a precursor lesion of PHAT(4). Groisman et al postulated that increased VEGF production and neovascularization could be due to hypoxia induced by hyalinized vessels(5,6).

A comprehensive literature search was conducted in the PubMed and Scopus databases to identify all published reports of pleomorphic hyalinizing angiectatic tumor (PHAT) from 1996 to date. The search strategy used the keywords “pleomorphic hyalinizing angiectatic tumor” AND/OR “PHAT” in the title or abstract. Articles were included if they reported individual cases or case series with adequate clinical, radiological, and histopathological details. Additionally, the reference lists of all retrieved articles were manually screened to identify any further relevant reports. Using this strategy, a total of approximately 133 cases were identified, which have been documented in the international literature to date. Data on patient demographics, tumor location, size, and histopathology were extracted and summarized (Table 1). Immunohistochemical studies, treatment, and outcomes, if any, were noted. The average age of presentation among the 133 cases was found to be 56 years (range: 10-86 years). In our series, the average age of presentation was 39.7 years. This tumor showed a female preponderance, with 56% (n=73 cases) being female and 44% (n=57 cases) being male. The gender in 3 cases could not be retrieved. Our series showed a male preponderance, with 75% of cases being male. The sample size in our series is small, which could account for the differences in average age and gender compared with the reported cases in the literature. The clinical presentation was typically a slow-growing, mostly painless, and occasionally painful mass, often causing functional discomfort. Bruising or hematoma formation may also be observed(4,6). The duration of symptom presentation varied widely, ranging from 2 months to 30 years, with an average of 3.4 years, consistent with two cases in our series. Some previously reported cases were also detected incidentally. The most common sites of presentation in descending order of frequency were found to be lower extremities (50%), upper extremities (8%), back and buttocks (5%), inguinal area and abdominal wall (3%), retroperitoneal region, breast, oral cavity, chest wall, kidney, and mesorectum (2%), larynx, neck, eyelid, nasal cavity, scrotum, and vulva (1%), and other sites (12%). Our series included one case each in the upper and lower extremities, consistent with the most commonly reported sites, and two cases on the abdominal wall, which is less commonly affected. Across reported cases, tumors measured an average of 7.9 cm in their greatest dimension, ranging from 1.5 cm to 25.6 cm. The mean maximum tumor size in our series was 6 cm.

**Table 1:** Summary of published PHAT cases in international literature

Sl No.	Author	No. of Cases	Age of Patients (Years)	Gender of Patients	Localization With Number of Cases
1	Smith (3)	14	59 (range: 32-83)	8 F, 6 M	Lower extremity, 9; other sites, 5
2	Silverman JS (1)	1	59	F	Right foot
3	Fukunaga M (4)	1	58	M	Axilla
4	Gallo C (1)	1	80	F	Right popliteal fossa
5	Brim SP (5)	1	-	-	Foot
6	Groisman GM (1)	2	41, 44	2 F	Lower extremity, 2
7	Husek K (4)	1	63	M	Forearm
8	Matsumoto K (4)	1	83	F	Left thigh
9	Iwamoto CA (1)	1	49	F	Axilla
10	Ide F (4)	1	86	F	Buccal mucosa
11	Folpe AL (1)	41	53 (range: 10-79)	22 F, 19 M	Lower extremity, 31; other sites, 9
12	Fujiwara M (4)	1	69	M	Back
13	Lin O (5)	1	45	F	Right medial knee
14	Lee JC (4)	1	63	M	Right breast
15	Lum D (5)	1	76	M	Right thigh
16	Luzar B (1)	1	47	F	Ankle region
17	Capovilla M (1)	1	66	M	Right buttock
18	El-Tal AE (4)	1	60	F	Right foot
19	Kazakov DV (4)	1	76	F	Axilla
20	Ke Q (1)	9	46 (range: 19-76)	5 F, 4 M	Lower extremity, 3; inguinal region, 2; waist, chest wall, forearm, and buttock, 1 each
21	Jaggon JR (1)	1	77	F	Left loin
22	Iascone C (5)	1	53	F	Left mesorectum
23	Tallarigo F (4)	1	75	M	Breast
24	Peng HC (1)	1	49	M	Right buttock
25	Cimino-Mathews A (4)	1	46	M	Right calf
26	Parameshwarappa S (1)	1	65	F	Upper limb
27	Labanaris AP (4)	1	68	M	Right scrotum
28	Mohajeri A (4)	2	58, 33	1 F, 1 M	Toe, knee
29	Sofos S (10)	1	62	M	Right anterior leg
30	Illueca C (1)	2	51	2 F	Back, palpebra
31	Choong MY (1)	1	53	M	Inguinal area
32	Subhawong TK (4)	3	46, 49, 87	2 F, 1 M	Lower extremity, 2; upper extremity, 1
33	Wei S (4)	1	37	F	Back
34	Idrees MT (3)	1	72	F	Renal hilum
35	Rekhi B (1)	1	63	F	Lower extremity
36	Kuang P (1)	1	35	F	Neck
37	Suzuki K (1)	1	68	F	Thigh
38	Changchien YC (4)	1	76	M	Upper extremity
39	Fan C (4)	1	51	F	Chest wall
40	Yorita K (7)	1	56	M	Right thigh
41	Raghavan V (5)	1	22	F	Right buttock
42	Felton SJ (4)	1	61	M	Back
43	Morency E (1)	1	55	F	Dorsum of foot
44	Onyemkpa C (8)	1	75	F	Groin
45	Brazio PS (4)	1	22	F	Forearm
46	Kane PM (1)	1	35	M	Hand
47	Chu ZG (4)	1	26	F	Retroperitoneum
48	Chalmeti A (6)	1	50	M	Left calf
49	Scalici Gesolfo C (1)	1	61	F	Kidney
50	Jaramillo CJ (4)	2	50, 72	2 M	Right buttock, 2
51	Braiki M (11)	1	40	F	Pelvic retroperitoneum
52	Sadeghi A (12)	1	43	M	Right ankle
53	Szep Z (1)	1	63	M	Left crura
54	Balasubiramaniyan V (1)	1	30	F	Mesorectum
55	Hammouda SB (13)	1	50	M	Abdominal wall
56	Yamamoto A (5)	1	74	F	Right popliteal fossa
57	Salim AA (4)	1	61	M	Breast
58	Kökoğlu K (3)	1	33	F	Oral cavity
59	Cazzato G (1)	1	48	F	Thigh
60	da Silva EC (5)	1	83	F	Vulva
61	Salehipour M (9)	1	41	M	Retroperitoneum
62	Martínez A (3)	1	70	F	Back
63	Zaninovich AT (14)	1	-	-	Larynx
64	Neblett C (15)	1	41	F	Lower back
65	Sun S (16)	1	32	M	Nasal cavity
66	Kini (17)	1	65	M	Abdominal wall

Radiology typically shows a well-defined soft tissue mass; MRI is more sensitive, revealing edema, hemorrhage, and characteristic T1/T2 signal patterns. These tumors may show infiltrative margins with prominent ectatic arterial vessels(4). Radiologic assessment of the two cases in our series indicated that both lesions were benign soft tissue tumors.

Fine needle aspiration cytology has diagnostic pitfalls due to nonspecific and overlapping features, making histopathology essential to avoid misdiagnosis(6,7).

Grossly, these tumors typically appear as solid, lobulated masses with a tan to maroon coloration. Although encapsulation is generally absent, the lesions are usually well demarcated but may exhibit locally infiltrative margins. Some tumors may also contain blood-filled cystic components. Microscopically, the tumors are unencapsulated and display alternating hypercellular and hypocellular areas. Hypercellular regions are composed of sheets and fascicles of spindle-shaped tumor cells. Pleomorphic cells, predominantly in the central portion of the lesion, exhibit single or multilobated nuclei, occasional nuclear pseudoinclusions, and moderate cytoplasm. Numerous variably sized ectatic blood vessels are present, often showing subendothelial fibrin deposition, luminal thrombi, and perivascular hyalinization. Areas of fresh hemorrhage or hemosiderin deposition are frequently observed adjacent to these vessels. The stroma is myxoid and contains a dense mixed inflammatory infiltrate. Mitotic activity is generally low despite pronounced cellular pleomorphism. At the periphery, the tumor may demonstrate an infiltrative pattern into surrounding adipose tissue. A few cases displaying glomeruloid or papillary endothelial hyperplasia have also been described(1,5,6).

Immunohistochemically, these tumors demonstrate positivity for CD34 (approximately 70% of cases), vimentin, and factor VIIIa (20%-40% of cases). VEGF expression is observed in endothelial cells of nonhyalinized vessels at the tumor periphery. CD99, ER, PR, and Bcl-2 expression has also been reported, although these markers have no diagnostic significance. The Ki-67 labeling index is generally low (<5%) and seen in the nuclei of the pleomorphic cells. The tumor cells are consistently negative for S100, desmin, SMA, SOX10, cytokeratin, CD117, STAT6, p63, and CD31(1,5). Ultrastructural studies demonstrate abundant cytoplasmic vimentin filaments within the neoplastic cells, indicative of fibroblastic differentiation(4,5).

The differential diagnosis could be varied and includes neurofibromas, schwannomas, undifferentiated pleomorphic sarcomas, malignant fibrous histiocytomas, solitary fibrous tumors, giant cell angiofibroma, and Kaposi sarcoma(4). Neurofibromas are S100 and EMA positive and typically lack ectatic vessels. Schwannomas may exhibit extensive hyalinization, ectatic vessels, and nuclear pseudoinclusions; however, they do not have infiltrative margins and are S100 positive(1). Undifferentiated pleomorphic sarcomas and malignant fibrous histiocytomas have higher mitotic activity, do not show intranuclear inclusions, and are negative for CD34(1,5). Solitary fibrous tumors are characterized by hemangiopericytoma-like vasculature within a hyalinized stroma. Kaposi sarcoma demonstrates proliferation of small vessels surrounding ectatic channels and is CD31 positive.

PHAT shows both morphologic and genetic overlap with hemosiderotic fibrolipomatous tumor (HFLT) and myxoinflammatory fibroblastic sarcoma (MIFS). HFLT differs from PHAT in that it is composed of mature adipocytes separated by fibrous septa containing spindle cells with mild atypia. Intranuclear inclusions and hemosiderin-laden macrophages are absent. MIFS exhibits Reed-Sternberg-like giant cells and virocytes with bizarre nuclei and macronucleoli. In our series of four cases, microscopy did not reveal any areas suggestive of HFLT or MIFS. Flow cytometric studies have identified balanced translocations between chromosomes 1 and 3 and chromosomes 1 and 10 mapped to transforming growth factor-β receptor 3 (TGFBR3) and meningioma-expressed antigen 5 (MGEA5)(4). The percentage of translocation varies in PHAT, MIFS, and HFLT(2).

The treatment of choice for PHAT continues to be wide surgical excision with histologically confirmed negative margins. Recurrence rates, however, range from 30% to 50%, with a mean time to recurrence of 3.6 years(1,8,9). In our case series, no recurrences were observed among the four cases, with two patients followed for 3 years and two patients for 2 years. Adjuvant local radiotherapy has been suggested to reduce the risk of recurrence(1,4). The use of chemotherapy is generally not indicated, as these tumors rarely metastasize(5). Although PHAT is typically considered locally aggressive, progression to myxofibrosarcoma, which carries metastatic potential, has been reported in three cases(8). Therefore, long-term follow-up is recommended for all patients with PHAT.

## Conclusion

PHAT is a rare tumor with low- to intermediate-grade malignant potential, with about 133 cases reported in the international literature. Through this study, we aim to contribute to the existing literature and raise awareness of this rare entity, which shares histological similarities with a broad spectrum of benign and low-grade malignant tumors. Accurate histopathological diagnosis is essential to guide appropriate management and follow-up.

## Data Availability

The manuscript contains all of the data and materials used.
